# Predictors of congenital anomalies among newborns in Arsi zone public hospitals, Southeast Ethiopia: a case-control study

**DOI:** 10.1186/s13052-021-01093-6

**Published:** 2021-06-30

**Authors:** Sudi Jemal, Engidaw Fentahun, Mohammed Oumer, Abebe Muche

**Affiliations:** Department of Human Anatomy, School of Medicine, College of Medicine and Health Sciences, University of Gondar, Gondar, Amhara Ethiopia

**Keywords:** Congenital anomalies, Pregnancy, Predictors, Folic acid, Pesticides, Alcohol and khat

## Abstract

**Background:**

Congenital anomaly is a partial or complete structural and/or functional defect during intrauterine life. Globally, major congenital anomalies account for 6% of all newborns among which about 94% of cases occurred in developing countries. In spite of its public health importance, very limited studies are reported in Ethiopia, and hardly any study in Arsi Zone.

**Objectives:**

To determine the predictors of congenital anomalies among newborns in Arsi Zone Public Hospitals, Southeast Ethiopia.

**Methods:**

A multi-center institutional-based case-control study was conducted in 418 (105 cases and 313 controls) of newborns in Arsi Zone Public Hospitals. Descriptive analysis, binary and multivariable logistic regressions were implemented.

**Results:**

In this study, women who have been drinking alcohol during pregnancy were 3.48 times more prone to have newborns with congenital anomalies than their counterparts (AOR = 3.48; 95% CI: 1.38, 8.74). The likelihood of having a newborn with congenital anomalies was six and four times higher for women who had a maternal illness (AOR = 6.10; 95%CI: 2.39, 15.57) and chewing khat during pregnancy (AOR = 4; 95%CI: 1.49, 10.65), respectively. Moreover, the lack of folic acid supplementation and pesticides during pregnancy were 3.25 and 4.76 times more likely to experience a newborn with congenital anomalies, respectively.

**Conclusion:**

Alcohol drinking, maternal illness, khat chewing, and chemical exposure during pregnancy had a significant association with the occurrence of congenital anomalies. While, taking folic acid supplements had a protective effect. Health experts and the community should take these factors into consideration and act accordingly.

## Background

Congenital anomalies (CA) are partial or complete structural and/or functional developmental disorders during intrauterine life and may be appreciated at birth or later in life [1]. It can also be described as congenital malformation or birth defects. The risk of congenital anomalies is high during the embryonic period (3rd to 8th week of gestational age) which is the critical period for the development of the fetus. Thus, a factor that interferes with the process of the organogenesis during this sensitive period, presumably, increases the risk for CA. Due to various factors including, environmental factors, chemical agents, radiation, drugs, maternal lifestyle, infections, nutrition, or combinations of these factors, congenital anomalies can happen at the level of each organ or organ system.

Worldwide, major congenital anomalies account for 6% of all newborns among which about 94% of these defects occur in developing countries. CA contributes to 20–30% of infant mortality [2] and 20% of stillbirth [3]. According to 2015 World health statistics, globally about 303,000 newborns die due to congenital anomalies before they reach 1 month each year [4]. In Europe, the average infant mortality due to congenital anomaly was 1.1 per 1000 births [5]. Moreover, in addition to stillbirth and neonatal death, congenital anomalies are the main causes for survivors’ lifelong mental and physical disabilities that may have significant impacts not only on the community but also on individuals and family in particular [4, 6].

According to WHO reports, removal of risk factors and reinforcement of certain protective strategies reduces the occurrence of congenital anomalies. These include avoiding teratogen substances and other environmental factors, controlling and treating maternal illnesses, screening for infections and treatment, supplying mothers with adequate healthy diets and vitamin and folic acid supplementations, and vaccination for children and mothers [4]. In developed countries, safeguarding the exposure of women to teratogen prior to conception and/ or during the early stage of pregnancy was found to be a key strategy to control the occurrence of about 70% of congenital anomalies [6].

In Africa, the prevalence of congenital anomalies ranges from 5.2 to 74.5 per 10,000 births [7]. Amazingly, about 94% of severe congenital anomalies occur in low-and middle-income countries [4], of which about 190,000 babies delivered each year with neural tube defects [8]. A study done in Cote d’Iviore revealed that CA accounts for 52% of the mortality rate. Of these, gastroschisis is the leading lethal disease with 100% mortality [9]. In Ethiopia, the overall prevalence of congenital anomalies was about less than approximately 2% [10–12] of which 40.3% is attributable to neural tube defect [10] which was lower than other African studies [13, 14].

Generally, in Ethiopia there were insufficient studies conducted on congenital anomalies and associated risk factors, studies indicated regional variation on the occurrence of congenital anomalies [10, 15, 16]. Therefore, since Ethiopia is the home to many communities with variable characteristics, it is important to conduct research in different regions among different communities. As far as our search is concerned, even though there are some studies conducted in different parts of the country, there is no prior study on CAs conducted in Arsi Zone, Southeast Ethiopia. Hence, the present study aims to assess the predictors of congenital anomalies in the setting.

## Methods and materials

### Study area and period

The multi-center institutional-based case-control study design was conducted among newborns in Arsi zone public hospitals from December 01, 2020 to May 30, 2020. The study was conducted in Arsi Zone Public Hospitals. Arsi Zone is located in Oromia Regional State Southeastern part of Ethiopia. The capital city of Arsi zone is Asella, which is 174 km away from Addis Ababa, the capital city of Ethiopia. The hospitals are Asella Referral Hospital, Bekoji, Abomsa, Robe, Gobessa, Kersa, Sude, and Bale District Hospitals.

### Source and study population

The source population is all newborns delivered in Arsi Zone Public Hospitals. The study population is all newborns delivered in Arsi Zone Public Hospitals and fulfill the inclusion criteria during the study period.

### Sample size determination and sampling procedures

The sample size was calculated by using Fleiss with continuity correction factor formula with a case-control ratio of 1:3; power of 80%, a significance level of 5%, the proportion of controls exposed (alcohol drinking) 55% [16], assuming the minimum odds ratio to be detected was 2. Using this value the expected percent of exposure among cases was 70%. The computed sample size was 418 (105 cases, and 313 controls).

All public hospitals found in the Arsi zone were included and an equal proportion allocation of samples was performed to include participants from each hospital. All mothers who delivered infants with congenital anomalies were invited to participate in the study. Mothers who delivered infants with no congenital anomalies were also included in the study using a systematic random sampling procedure. In the present study, all newborns with visible congenital anomalies and the next three newborns (delivered after cases) without congenital anomalies were included.

### Study variables

In our study, congenital anomalies are considered as the dependent variable. On the other hands, Socio-demographic characteristics (age, educational status, marital status, religion, ethnicity, income, occupation, husband’s education, and occupation), obstetric history (folic acid during and prior to pregnancy, history of antenatal care (ANC), contraceptive use, parity, gravidity, gestational age, family history of congenital anomalies, previous history of a child with congenital anomalies), medical condition-related factors (unidentified medication use, medical illness), drug and radiation (smoking and alcoholic history, khat chewing, use of herbal medicines, radiation, exposure to chemicals like pesticides), and others like sex of the newborn, consanguinity are considered as independent variables.

### Operational definition

Cases:- are mothers who gave birth to babies with externally visible defects to any system of the body identified by clinical examinations by experienced medical doctors at the time of delivery.

Controls:- are mothers who gave birth babies without externally visible defects to any system of the body after clinical examination by experienced medical doctors at the time of delivery.

Consanguineous marriage:- couples related by blood at least to the second cousin.

Radiation:- Exposure to x-ray.

### Data collection procedure

Data were collected using a pre-tested structured interviewer-administered questionnaire, which includes questions of socio-demographic, obstetric, medical, alcohol intake and smoking history, use of herbal medicine, exposure to chemicals, radiation, and folic acid supplementation (5 mg of folic acid was given throughout first trimesters from conception to 12th week of pregnancy). The questionnaire was prepared in English then translated into Amharic and Afan Oromo and then back to English by a third party who is native in Amharic and Afan Oromo respectively and good in English to ensure consistency of translation. Newborns were carefully examined and assessed for congenital anomalies by experienced medical doctors. Following a proper medical examination, the data were gathered from mothers regarding the information explained above using a structured questionnaire.

Six data collectors and six supervisors who are fluent in Amharic and Afan Oromo were involved in data collection. Accordingly, the supervisors were supervised respective data collectors during the data collection time and receive the collected and crosschecked questionnaires from them. Finally, the supervisors reported and discussed and crosschecked data with the investigator on a daily basis throughout the data collection period.

### Data quality assurance

To maintain data quality, data collectors who are fluent in Amharic and Afan Oromo were selected based on their educational level and experience in data collection. Detail orientation and training about data collection and the purpose of the study were given to supervisors and data collectors by the principal investigator. Based on previous related peer-reviewed published studies, the questionnaire were developed by the investigators. A pretest study was conducted at Halila health centers on fifty individuals to see for validity and reliability of tools. The collected data were reviewed and checked for mistakes, completeness and consistency by the investigators and supervisors on a daily basis during data collection.

### Data management and analysis

The collected data were coded, checked, and entered using Epi-Info version 7.0 and exported to IBM SPSS Version 21 for cleaning, checking for missing values, and analysis of data. Descriptive statistics and frequency tables were used to describe study populations.

Binary and multivariable logistic regression analyses were employed to assess the degree of association between dependent and independent variables. Those variables associated in binary logistic regression with a significance value of *p*-value ≤0.2 were entered into a multivariable logistic regression to identify predictors of CAs. A p-value ≤0.05 with 95% CI was considered statistically significant to identify significant factors associated with congenital anomalies. Hosmer-Lemeshow goodness-of-fit test was used to assess the fitness of the model.

## Results

### Socio-demographic characteristics of the study participants

A total of 418 (105 cases and 313 controls) mothers were successfully interviewed at the labor ward after delivery of a child with a response rate of 100%. About 84.7% of controls and more than half of cases (66.7%) were in the age group of 20–34. Nearly above half of the women among cases (54.3%) and most of the women among controls (77.6%) were married as presented in Table 1. The majority of women (54.3% of cases and 46% of controls) were Muslims in religion. Around 44.8% of cases and 42.2% of controls were illiterate. Almost half of the women.

**Table 1:** The socio-demographic characteristics of the study participants in Arsi Zone Public Hospitals, Southeast Ethiopia, 2020

Characteristics	Categories	Case N (%)	Control N (%)
Age of mother	< 20	8(7.6)	13(4.2)
	20–34	70(66.7)	265(84.7)
	> 34	27(25.7)	35(11.2)
Marital status of mothers	Married	57(54.3)	243(77.6)
	Divorced	17(16.2)	27(8.6)
	Widowed	8(7.6)	11(3.5)
	Single	17(16.2)	21(6.7)
	Separated	6(5.7)	11(3.5)
Religion of mothers	Muslim	57(54.3)	144(46)
	Orthodox	28(26.7)	126(41.2)
	Protestant	28(8.9)	16(15.2)
	Catholic	12(3.8)	4(3.9)
Educational level of mothers	No formal education	47(44.8)	132(42.2)
	Primary education	24(22.9)	68(21.7)
	Secondary education	19(18.1)	56(17.9)
	Higher education	15(14.3)	57(18.2)
Occupation of mothers	Housewife	52(49.5)	139(44.4)
	Government employee	4(3.8)	31(9.7)
	Private business	22(21)	89(28.4)
	NGO employee	13(12.4)	21(6.7)
	Student	14(13.3)	33(10.5)
Educational level of fathers	No formal education	44(42.3)	125(39.9)
	Primary education	28(26.9)	58(18.5)
	Secondary education	21(20.2)	57(18.2)
	Higher education	11(10.6)	73(23.3)
Residence	Urban	53(50.5)	165(52.7)
	Rural	52(49.5)	148(47.3)
Ethnicity	Oromo	71(67.6)	185(59.1)
	Amhara	26(24.8)	93(29.7)
	Tigre	6(5.7)	15(4.8)
	Gurage	2(1.9)	18(5.8)
	Others	0(0)	2(0.6)

(50.5% of cases and 52.7% of controls were urban dwellers. Concerning the ethnicity of mothers, the majority of cases (67.6%) and controls (59.1%) were Oromo.

### Reproductive and obstetric characteristics

As it is presented in Table 2, the majority of women (81% of cases and 75.1% of controls) were multigravida. Almost half of the controls (52.1%) and 41.9% of cases were born at second order. Fifty-six percent of cases and 74% of controls were born at 37 weeks and above and a small proportion of both cases (2.9%) and controls (2.9) were born prior to 28 weeks. Nearly equal proportion of cases (5.7%) and controls (5.4) had a previous abortion history. Regarding family history and previous history of children with congenital anomalies, less than 20% of both cases and controls reported a family history and previous history of a child with congenital anomalies. This study revealed that the number of females with congenital anomalies (60%) were higher than those without congenital anomalies (38.7%). About 41% of mothers of children with congenital anomalies and 55% of mothers of children without congenital anomalies were taken folic acid supplementation in the early period of pregnancy. Similarly, contraception use around conception was 59% in mothers who had children with congenital anomalies and 44.7% of mothers who had children without congenital anomalies.

**Table 2:** Reproductive and obstetric characteristics of study participants in Arsi Zone Public Hospitals, Southeast Ethiopia, 2020

Characteristics	Categories	Case N (%)	Control N (%)	Total N (%)
Gravidity	Primigravida	20(19)	78(24.9)	98(23.4)
	Multigravida	85(81)	235(75.1)	320(76.6)
Birth order	First	20(19)	78(24.9)	98(23.4)
	Second	44(41.9)	163(52.1)	207(49.5)
	Third	27(25.7)	41(13.1)	68(16.3)
	Above third	14(13.3)	31(9.9)	45(10.8)
Gestational age at birth	< 28 weeks	3(2.9)	9(2.9)	12(2.9)
	28–37	43(41)	72(23)	115(27.5)
	> 37 weeks	59(56.2)	232(74.1)	291(69.6)
Stillbirth	Yes	12(11.4)	16(5.1)	28(6.7)
	No	93(88.6)	297(94.9)	390(93.3)
Abortion	Yes	6(5.7)	17(5.4)	23(5.5)
	No	99(94.3)	296(94.6)	395(94.5)
Family history of congenital anomalies	Yes	17(16.2)	55(17.6)	72(17.2)
	No	88(83.8)	258(82.4)	346(82.8)
Previous history of child with congenital anomalies	Yes	17(16.2)	48(15.3)	65(15.6)
	No	88(83.8)	265(84.7)	353(84.4)
Sex of newborn	Male	42(40)	192(61.3)	234(56)
	Female	63(60)	121(38.7)	184(44)
ANC follow-up	Yes	49(46.7)	146(46.6)	195(46.7)
	No	56(53.3)	167(53.4)	223(53.3)
Folic acid supplementation	Yes	43(41)	172(55)	215(51.4)
	No	62(59)	141(45)	203(46.8)
Contraceptive use	Yes	62(59)	140(44.7)	202(48.3)
	No	43(41)	173(55.3)	216(51.7)

### The type of congenital malformation

**Fig. 1:**
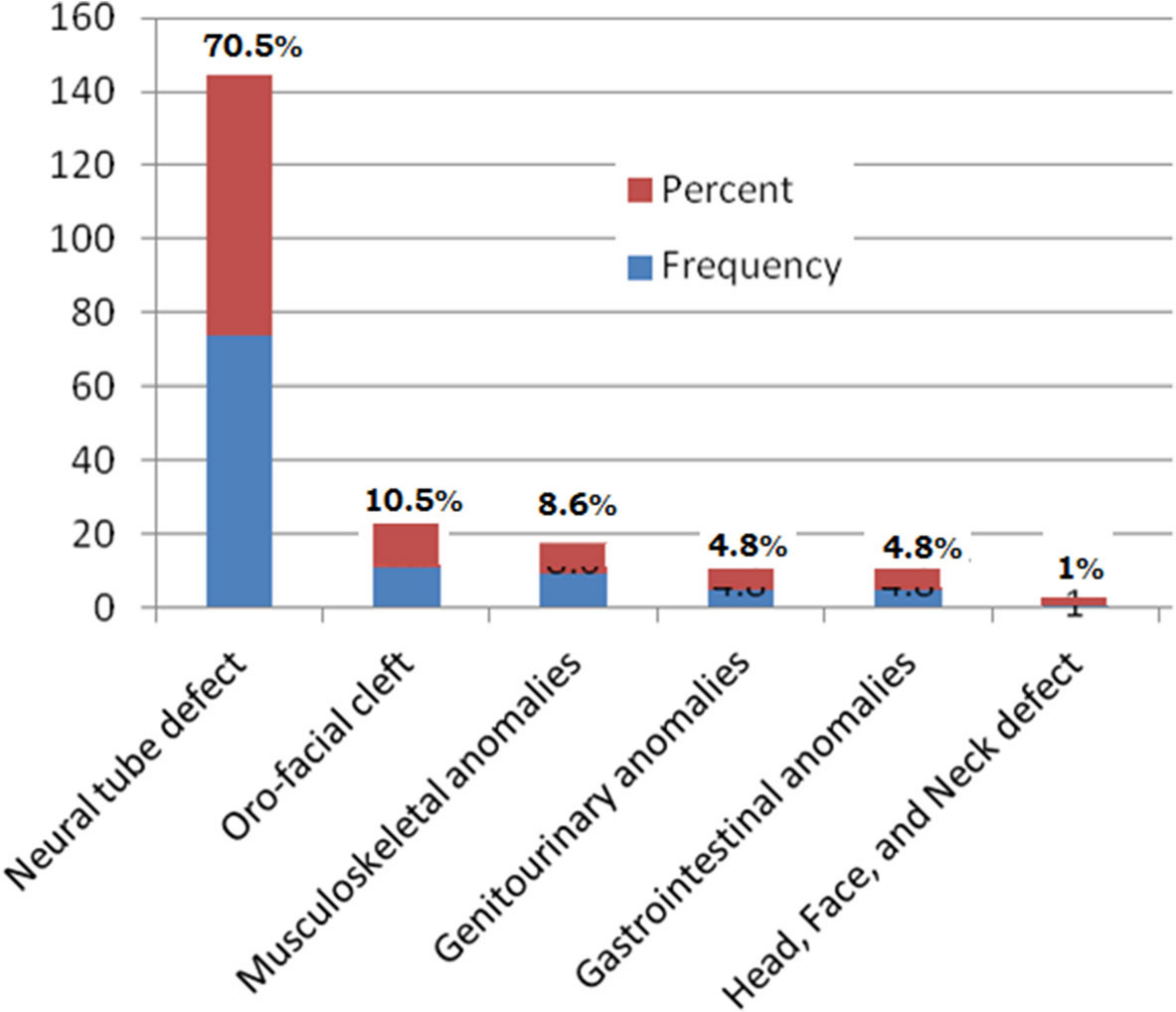
The precentage distribution of selected congenital anomalies in Arsi zone public hospitals, Southeast Ethiopia, 2020

Among 105 newborns presented with congenital anomalies, neural tube defects (70.5%) were the most manifested anomalies, succeeded by an orofacial cleft (10.5%) and musculoskeletal anomalies (8.6%). Out of the diagnosed anomalies, genitourinary and gastrointestinal anomalies were 4.8% (Fig. 1). Furthermore, the Percentage distributions of selected congenital anomalies by type of anomaly is presented in Table 3.

**Table 3:** Percentage distributions of selected congenital anomalies by type of anomaly, Arsi Zone Public Hospitals, Southeast Ethiopia, 2020

Type of Congenital anomalies	Frequency	Percent
**Spina bifida**	16	3.8
**Hydrocephalus**	11	2.6
**Anencephaly**	44	10.5
**Myelomeningocele**	3	0.7
**Meningocele**	4	1
**Cleft lip**	4	1
**Cleft palate**	2	0.5
**Cleft lip with palate**	5	1.2
**Club foot**	5	1.2
**Talipe equinovarus**	7	1.67
**Gasroschisis**	5	1.2
**Imperforated anus**	1	0.2
**Omphalocele**	4	1
**Hypospadia**	4	1
**Genital absence**	1	0.2

### Risk factors associated with congenital anomalies

In the present study, consanguineous marriage was reported in 58.1% of case mothers and 20% of control mothers (Table 4). About 64% of case mothers and 18% of control mothers have been drinking alcohol during their pregnancy period. Out of the study participants, maternal medical illness during pregnancy in mothers of newborns with and without congenital anomalies was 39 and 20.4%, respectively. About 67% of case mothers and 21% of control mothers were exposed to chemicals like pesticides at the time of pregnancy. Chewing khat and using other herbal medicines were 65.7 and 46.7% of mothers who had infants with congenital anomalies and 19.5 and 19.2% in mothers who had infants without congenital anomalies, respectively.

**Table 4:** The analysis of risk factors associated with congenital anomalies in Arsi Zone Public Hospitals, Southeast Ethiopia, 2020

Characteristics	Congenital anomaly		COR (95% CI)	AOR (95% CI)
	Case	Control		
	N (%)	N (%)		
**Consanguinity**				
Yes	61(58.1)	62(19.9)	5.59(3.47, 9.01)	1.74 (0.78,3.86)
No	44(41.9)	256(80.1)	1	1
**Drink alcohol**				
Yes	67(63.8)	57(18.2)	7.92(4.89,12.94)	3.48(1.38,8.74)*
No	38(36.2)	256(81.1)	1	1
**Smoking**				
Yes	58(55.2)	52(16.6)	0.16(0.1,0.26)	1.47(0.58,3.76)
No	47(44.8)	261(83.4)	1	1
**Maternal illness**				
Yes	41(39)	64(20.4)	2.5(1.54, 4.02)	6.10(2.39,15.57)*
No	64(61)	249(79.6)	1	1
**Unidentified drug use**				
Yes	50(47.6)	60(19.2)	3.8(2.38,6.17)	0.43(0.16,1.2)
No	55(52.4)	253(80.8)	1	1
**Chewing khat**				
Yes	69(65.7)	61(19.5)	7.92(4.85,12.93)	4(1.49,10.65)*
No	36(34.3)	252(80.5)	1	1
**Chemical exposure**				
Yes	70(66.7)	67(21.4)	7.34(4.51,11.96)	4.76(1.57,14.47)*
No	35(33.3)	246(78.6)	1	1
**Herbal exposure**				
Yes	49(46.7)	60(19.2)	3.7(2.3,5.94)	0.91(0.4,2.07)
No	56(53.3)	253(80.8)	1	1
**Lack of folic acid use**				
Yes	43(41)	172(55)	1.8(1.12,2.8)	3.25(1.6,6.61)*
No	62(59)	141(45)	1	1
**Contraceptive use**				
Yes	62(59)	140(44.7)	1.8(1.14,2.8)	1.16(0.51,2.65)
No	43(41)	173(55.3)	1	1

Key: * statistically significant at P-value ≤0.05 in multivariable logistic regression analysis

The binary logistic regression analyses (using crude odds ratio) displayed that consanguinity, alcohol drinking during pregnancy, smoking during pregnancy, maternal illness, history of drug use during the current pregnancy, khat chewing during pregnancy, exposure to chemicals during pregnancy, exposure to herbal during pregnancy, lack of folic acid supplementation during early pregnancy, and use of contraception around pregnancy were significantly associated with the occurrence of congenital anomalies. However, maternal age, educational level of the mother, parental occupation, residence, gravidity, birth order, family history of CAs, previous history of a child with CAs, ANC follow-up, stillbirth, and abortion were not statistically significant (Table 4).

Those statistically significant variables in the binary logistic regression analyses were entered into a multivariable logistic regression to identify the predictor variables associated with congenital anomalies. As a result, women who have been drinking alcohol during the current pregnancy were 3.48 times more prone to give birth to newborns with congenital anomalies than their counterparts (AOR = 3.48; 95% CI: 1.38, 8.74). Likewise, the likelihood of having newborns with congenital anomalies was six and four times higher for women who had a maternal illness during pregnancy (AOR = 6.10; 95% CI: 2.39, 15.57) and chewing khat during pregnancy (AOR = 4; 95% CI: 1.49, 10.65), respectively. Women who have been directly exposed to chemicals in work fields like pesticides during pregnancy were 4.76 times more likely to have newborns with congenital anomalies as compared to those who did not expose (AOR = 4.76; 95% CI: 1.57, 14.47). Besides, women who had not taken folic acid supplementation during early pregnancy were three times more likely (AOR = 3.25; 95% CI: 1.6, 6.61) to have newborns with congenital anomalies as compared to those who had taken folic acid supplementation (Table 4).

## Discussion

The main findings of this study are a notably statistically significant association of alcohol drinking, maternal chronic illness, khat chewing, and chemical exposure during pregnancy to the congenital anomalies. Besides, folic acid supplementation during the early period of pregnancy had a protective effect on the induction of congenital anomalies.

Our study showed that alcohol drinking of any amount during pregnancy increases the risk of occurrence of congenital anomalies by about three times as compared to its counterparts. This is in line with the previous studies conducted in Ethiopia [12, 15] and California [17]. On the other hand, a very recent case-control study conducted in Bale Zone, Southeast, Ethiopia indicated that drinking alcohol had hardly any significant association with the occurrence of congenital anomalies [18]. This might be due to the differences in drinking alcohol cultures. As alcohol is capable of transmitting through placental membranes, it causes direct effects to the organogenesis of the developing embryo and fetuses and leading to structural abnormalities [19–22].

Infants born from mothers who had a maternal illness during pregnancy were six times more likely to develop congenital anomalies compared to infants born to mothers who were free from maternal illnesses. Our finding is strongly supported by the study that dealt with the prevalence and associated factors of birth defects in Northwest Ethiopia [12]. Similarly, maternal illnesses, like febrile illness and chronic diseases like diabetes mellitus were reported as they were associated with the occurrence of congenital anomalies [23, 24]. Unlike current observation, however, other study done in Ethiopia revealed that maternal illness had a hardly significant association with the existence of an infant with congenital anomalies [15]. This difference might be due to a lack of knowledge on the definite time of the embryonic period at which maternal illness happened.

In this study, the mother who has been exposed to chemicals during the current pregnancy was 4.8 times more prone to have infants with congenital anomalies compared to their corresponding counterparts. Our finding is strongly supported by previous studies carried out by different researchers elsewhere [15, 18, 25–27].

A study conducted among Yemeni pregnant women and a case-control study among Southeast Ethiopian pregnant women revealed that khat chewing during the early period of pregnancy was 2.02 and 3 times more likely to deliver an infant with congenital anomalies compared to those who did not chew khat, respectively (50,51). Similarly, in the present study, khat chewing during pregnancy augmented the development of congenital malformation by four folds as compared to those non-khat chewing mothers.

In our study area, khat chewing is the commonest and most popular social practice in which most of the individuals, including pregnant women, were engaged. In addition, khat chewing had high cultural, traditional, and social values. Therefore, we emphasize that this highly popular, but harmful social activity was one of the biggest confrontations to the public health experts in Ethiopia. However, it is the one that public health experts and communities as general must deal with.

In the present study, the lack of folic acid supplementation had a significant association with the occurrence of congenital anomalies. Women who did not take folic acid supplementation during pregnancy had about 3.25 times more chances to have infants with congenital anomalies compared to their counterparts. Our finding is in line with studies done elsewhere reported that folic acid supplementation had a protective effect against congenital anomalies [10, 12, 15, 18, 28]. In spite of the valuable protective role of folic acid, in Ethiopia, the use and coverage for folic acid supplementation is very limited [29]. However, the Ethiopian Federal Ministry of Health establishes a policy that makes pregnant women have folic acid supplementation. The gap between well-established policy and lack of folic acid supplementation might be due to poor awareness of pregnant women and health professionals towards the use of folic acid supplementation.

The most prevalent congenital anomalies were neural tube defects followed by orofacial cleft and musculoskeletal anomalies. This finding is consistent with the previous studies conducted in Ethiopia [10, 12, 15, 30, 31].

### Limitation of the study

Although diversified study participants were included in the present study,hospital-based unmatched case-control study makes the result of our study less generalizable to the community. The present study did not take the terminated pregnancy/cases of CAs into account rather it considered only externally visible CAs which are identified by clinical examination at birth.

## Conclusions

The present study observed that alcohol drinking, chemical exposure, khat chewing, maternal illness was associated with the occurrence of congenital anomalies. Folic acid use during the periconceptional period was identified as it had a protective effect against the occurrence of congenital anomalies. However, in the study area, most of the women had a lack of awareness on the use of folic acid supplementation in the early period of pregnancy.

## Recommendations

We emphasize that due attention must be given by public health experts and communities on the risk factors of congenital anomalies and preventive strategies. Further, in order to clearly identify the incidence and its determinant factors, prospective cohort study starting from preconception to birth in the study area and other parts of the country should be conducted.

## Data Availability

N/A

## Abbreviations

ANC: Antenatal care
AOR: Adjusted odds ratio
CA: Congenital anomaly
COR: Crude odds ratio
SPSS: Statistical Package for the Social Sciences
